# The identification of intact HIV proviral DNA from human cerebrospinal fluid

**DOI:** 10.1016/j.neurot.2024.e00373

**Published:** 2024-05-14

**Authors:** Zhan Zhang, Monica D. Reece, Sebastian Roa, William Tyor, Donald R. Franklin, Scott L. Letendre, Vincent C. Marconi, Albert M. Anderson, Christina Gavegnano

**Affiliations:** a Department of Pathology and Laboratory Medicine, Emory University School of Medicine, Atlanta, GA, USA; b Atlanta VA Medical Center, Atlanta, GA, USA; c Department of Neurology, Emory University School of Medicine, Atlanta, GA, USA; d Department of Psychiatry, University of California at San Diego School of Medicine, La Jolla, CA, USA; e Department of Medicine, Division of Infectious Diseases, University of California at San Diego School of Medicine, La Jolla, CA, USA; f Division of Infectious Diseases, Emory University School of Medicine, Atlanta, GA, USA; g Department of Global Health, Rollins School of Public Health, Emory University, Atlanta, GA, USA; h Emory Vaccine Center, Emory University, Atlanta, GA, USA; i Department of Pharmacology and Chemical Biology, Emory University School of Medicine, Atlanta, GA, USA; j Center for the Study of Human Health, Emory College, Atlanta, GA, USA; k Harvard Medical School, Center for Bioethics, Boston, MA, USA

**Keywords:** HIV, Reservoir, Central nervous system, Cerebrospinal fluid, Inflammation

## Abstract

We evaluated the HIV-1 DNA reservoir in peripheral blood mononuclear cells (PBMC) and cerebrospinal fluid (CSF) in people with HIV (PWH) and associations to cognitive dysfunction. Using the intact proviral DNA assay (IPDA), an emerging technique to identify provirus that may be the source of viral rebound, we assessed HIV DNA in CSF and PBMC in PWH regardless of antiretroviral therapy (ART). CSF was used as a sampling surrogate for the central nervous system (CNS) as opposed to tissue. IDPA results (3′ defective, 5′ defective, and intact HIV DNA) were analyzed by compartment (Wilcoxon signed rank; matched and unmatched pairs). Cognitive performance, measured via a battery of nine neuropsychological (NP) tests, were analyzed for correlation to HIV DNA (Spearman's rho). 11 CSF and 8 PBMC samples from PWH were evaluated both unmatched and matched. Total CSF HIV DNA was detectable in all participants and was significantly higher than in matched PBMCs (p ​= ​0.0039). Intact CSF HIV DNA was detected in 7/11 participants and correlated closely with those in PBMCs but tended to be higher in CSF than in PBMC. CSF HIV DNA did not correlate with global NP performance, but higher values did correlate with worse executive function (p ​= ​0.0440). Intact HIV DNA is frequently present in the CSF of PWH regardless of ART. This further supports the presence of an HIV CNS reservoir and provides a method to study CNS reservoirs during HIV cure studies. Larger studies are needed to evaluate relationships with CNS clinical outcomes.

## Introduction

A better understanding of HIV persistence during suppressive combination antiretroviral therapy (ART) is needed given the goal of ending the HIV epidemic. Suppression of HIV to levels of <50 copies/milliliter is achievable in most people with HIV (PWH) using current ART regimens [1]. However, HIV is not eradicated with effective ART [2]. Viral persistence results from the latency that is established shortly after infection, which is known to occur in CD4^+^ T-lymphocytes [3,4]. As a result, cure of HIV has been extremely elusive [5,6]. Furthermore, many antiretrovirals (ARVs) have much lower ARV concentrations in cerebrospinal fluid (CSF) compared to plasma [7]. Dolutegravir has been shown to achieve blood-brain-barrier (BBB) penetration with a CSF concentration comparable to plasma [8]. This trend of poor BBB penetrative ability in ARVs presents an opportunity for active viral replication and reservoir expansion in the brain.

The central nervous system (CNS) has emerged as an important HIV reservoir based on an expanding body of evidence. HIV DNA is commonly found in brain tissue from deceased PWH on suppressive ART [[9], [10], [11]]. Extensive research support that HIV can productively infect microglia [12], and integrated HIV DNA can be detected in astrocytes from brains of PWH despite virologic suppression [13]. Since many CNS cells such as astrocytes and microglia are long-lived, the HIV reservoir in the CNS may be particularly resistant to decay over time [14]. Viral genetic compartmentalization occurs in the brains of PWH and provides more support for an HIV CNS reservoir [15]. Assessing the HIV CNS reservoir has been limited to examination of autopsy brain tissues, highlighting a clinical need for a method to characterize HIV impact in the CNS for living PWH.

CSF, which traverses the BBB, provides one window into CNS HIV persistence, and is commonly sampled via lumbar puncture. HIV RNA becomes detectable in the CSF approximately eight days after the estimated date of transmission [16], indicating early CNS entry. The transactivator of transcription (Tat) HIV protein is detectable in CSF from 36.8% of individuals on suppressive ART [17]. Given its relatively short half-life, the presence of Tat indicates active HIV protein translation in the CNS. HIV persistence in the CNS has also been demonstrated by multiple reports of CSF viral escape despite peripheral viral suppression [18,19]. HIV DNA in the setting of virologic suppression has been found in CSF cells, and this presence of HIV DNA is associated with neurocognitive impairment, showing the clinical significance of HIV CNS persistence [20,21]. Innovative new techniques such as HIV Long Terminal Repeat sequencing have yielded detectable HIV DNA and HIV cell-associated RNA in >80% of CSF samples from PWH on ART [22]. This type of HIV CNS persistence is associated with higher neuronal damage by terminal continuation RNA amplification and the presence of cognitive dysfunction [23].

In a recently published study, the intact proviral DNA assay (IPDA) was successfully used to quantify latent HIV DNA from postmortem brain tissue. Specifically, intact HIV DNA by IPDA was identified from frontal lobe in six of nine individuals who were virologically suppressed on ART [10]. Ideally, the IPDA could be performed with CSF samples given the need to evaluate the CNS HIV reservoir over time as well as in cure studies. We hypothesized that HIV DNA could be detected in the CSF of PWH and would be associated with increased neurocognitive dysfunction. Therefore, we performed an evaluation of the IPDA using cryopreserved CSF cell pellet samples compared to peripheral blood IPDA and then examined associations between IPDA results and neuropsychological (NP) performance. To our knowledge, these data represent the first time that reservoir quantification in the CSF via IPDA has been linked to distinct neurocognitive deficits. These data address a major unmet clinical need toward identifying the role of the persistent HIV-1 reservoir in the CNS and its role in modulation of neurocognitive deficits and decline. The novelty of these data underscores the importance of conducting larger studies to elucidate the important relationship between the CNS reservoir, neurocognition, and eventual therapeutic interventions that may mitigate or reverse cognitive deficits in PWH.

## Methods

PWH were recruited from the Infectious Disease Program (IDP) Ponce de Leon Center, which is a comprehensive HIV clinic within the Grady Health System in Atlanta, Georgia. All PWH were diagnosed with HIV and receiving care at the IDP. Cryopreserved samples from adult PWH, regardless of ART status, enrolled in CNS studies (Emory IRB 00103851 – *Viral immunocapture and isolation of virus from CSF lymphoid and myeloid cells to better understand HIV in the central nervous system* [24]; Emory IRB 00039444 – *Novel tools for the assessment of patients with HIV-associated cognitive impairment: an observational study*) performed at the Emory Center for AIDS Research (CFAR) were used. Exclusion criteria for these studies were: 1) a history of severe neurologic and psychiatric diseases that are known to affect memory (including stroke, malignancy involving the brain, traumatic brain injury, schizophrenia, and AIDS-related opportunistic infections of the central nervous system); 2) active addictive substance use (cocaine, heroin, methamphetamine, or other non-cannabis addictive drug use in the last 30 days; cannabis use was permitted but participants were asked to abstain for 48 ​h prior to testing); and 3) heavy alcohol consumption in the last 30 days (defined as >7 drinks per week for women and >14 drinks per week for men). Clinical records were reviewed for substance abuse disorders and other psychiatric and neurological diagnoses. The 30 day abstinence period of non-cannabis addictive substance use was chosen to reduce subacute to chronic changes in the brain during chronic use and after clearance. Substance use abstinence was a historical evaluation. Given participant age range (36–68, median ​= ​53), it is unlikely that neurodegenerative diseases like Alzheimer's would affect the study population, interfering with NP assessments.

A battery of NP tests was administered that included nine tests used commonly in studies of cognition and HIV [25]: 1) Trailmaking Part A, 2) Trailmaking Part B, 3) Hopkins Verbal Learning Test-Revised (HVLT-R) total learning, 4) HVLT-R delayed recall, 5) Grooved Pegboard (dominant), 6) Grooved Pegboard (non-dominant), 7) Stroop Color Naming, 8) Stroop Color-Word interference, and 9) Letter Fluency (Controlled Oral Word Association Test). 7 domains were tested: executive functioning (tests 2,8), memory (test 4), motor (tests 5,6), learning (test 3), processing speed (tests 1,7), attention (test 8), and fluency (test 9) [26].

Scores were adjusted for demographic characteristics (including age, biological sex, race, and education) using published norms [[27], [28], [29]]. A composite global mean T score (NPT-9) was then calculated by averaging individual test T scores (49.76 ​± ​8.51). Global and domains were examined.

**Fig. 1:**
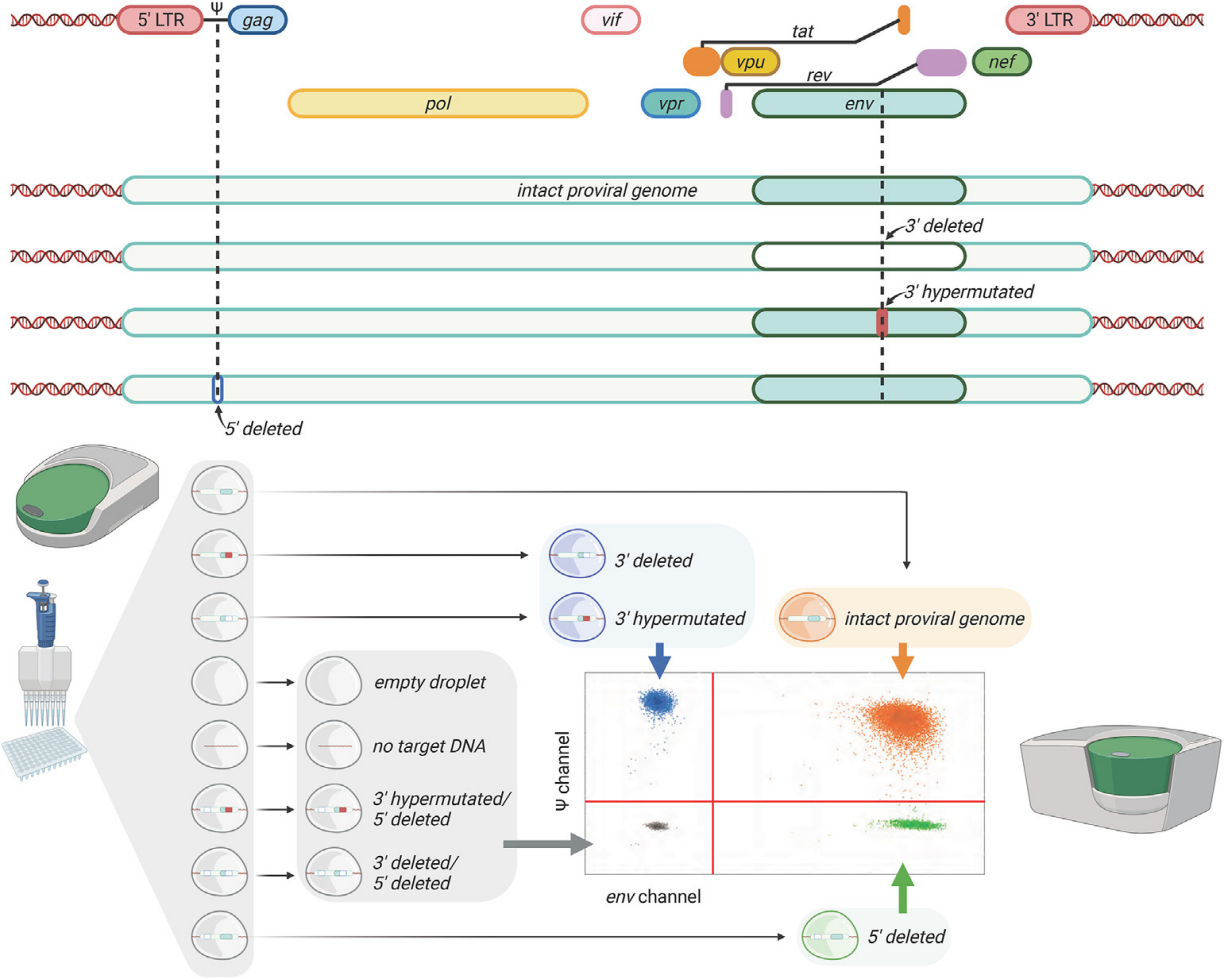
Schematic overview of Intact proviral DNA assay (IPDA). Top half: Illustration of IPDA-targeted defective regions of HIV-1 Ψ and Env. Bottom half: Multiplex polymerase chain reaction (PCR)-based 2-dimensional plot allows for discrimination of intact proviral DNA from defective.

This study was cross-sectional. Blood and CSF samples were obtained via veni and lumbar puncture at the same morning study visit. CSF was processed as per Emory CFAR and HIV Neurobehavioral Research Program (HNRP) protocols. Briefly, 10–25 ​mL (ml) of CSF were centrifuged at 300 ​g for 15 ​min. Peripheral Blood Mononuclear Cells (PBMC) samples were prepared as previously described [30]. After removal of supernatant, fetal bovine serum and dimethyl sulfoxide were added, and the resuspended pellets were then stored in a liquid nitrogen freezer. Cryopreserved cells from CSF and (when available) matched PBMC were thawed in a 37 ​°C water bath. Next, cells were immediately transferred to pre-warmed sterile Roswell Park Memorial Institute media. After counting and spinning down the cells, genomic DNA was extracted using the Gentra Puregene Cell kit (Qiagen). The frequency of IPDA was determined by duplex droplet digital polymerase chain reaction (ddPCR) (Fig. 1) as validated and published by Brunauer et al. [31] The multiplex PCR was performed on the Bio-Rad QX200 Digital Droplet PCR system. Up to 100 ​ng (ng) of genomic DNA extracted from CSF cell pellets, and 700 ​ng of genomic DNA extracted from PBMC were added to the master mix containing 2× ddPCR supermix (no dUTP, Bio-rad), primers (final concentration 900 ​nM), probes (final concentration 250 ​nM) and nuclease free water. Primer and probe sequences (5′ and 3’) are listed in Table S1 [31,32]. To ensure consistent PCR results, each sample was quantified in up to six replicates. PCR reaction droplets were prepared with the QX200 Droplet Generator. The thermal cycling program used for ddPCR reactions is in Table S2. The ddPCR plate was stored for at least 4 ​h at 4 ​°C prior to transfer to the QX200 droplet reader, a step which improves the quality metrics of droplets and achieves higher accepted droplet numbers per reaction. To correct for the frequency of total intact proviruses based on the DNA shearing index (DSI) during intact proviruses quantification, two sets of primers were designed for amplification of the human RPP30 gene (primers and probes listed in Table S3). The two regions of RPP30 gene that are targeted during multiplex PCR are spaced at the same distance as the Ψ and *env* amplicons. A previous study verified that DSI was comparable between RPP30 (RPP30-1 and RPP30-2) and HIV (Ψ and env) at all levels of shearing [31]. The Gentra Puregene Cell kit (Qiagen) was also used to limit the sheared DNA rate, and lowest DSI was 0.08.

Using two amplicons and hypermutations discrimination probes, the intact (Ψ+env+) and defective proviruses (Ψ+env- or Ψ-env+) were distributed into different quadrants (Qs) of the 2-dimensional amplitude ddPCR plots (Fig. 1). Droplets in Q1 contain proviral genomes including an APOBEC-3G-edited hypermutation or 3′ deletion and gave out a fluorescence signal (FAM) arising from an intact Ψ amplicon amplification. Q4, diagonally opposite to Q1, represents an intact *env* region with a deletion toward the 5’ encompassing Ψ region. Droplets that contain the intact proviral genome are located in Q2. Droplets that contain double defective regions or are not occupied with target proviral genomes are distributed in Q3.

For the statistical analyses, HIV DNA was quantified as copies per 10^6^ PBMC. Normality was assessed using the Shapiro-Wilk test. Given that the HIV DNA results did not meet assumptions of normality, the comparison between HIV DNA levels by compartment was performed with Wilcoxon signed rank for matched pairs and Wilcoxon rank sum for non-matched pairs. Correlations between HIV DNA and NP scores were performed with Spearman's rho. Statistical analyses were performed with SAS JMP (Cary, NC) as well as GraphPad Prism v10.0.2 (San Diego, CA). The study was approved by the Emory University Institutional Review Board and all participants provided informed consent.

## Results

There were 11 PWH (median age ​= ​53 years) with a long history of HIV (median estimated duration ​= ​138 months), see Table 1. Eight were men, two were women, and one was transgender man to woman. With respect to comorbidities, five had hypertension, four had depression, and two had chronic hepatitis B virus infection. Seven participants were on stable ART for at least six months with plasma and CSF HIV RNA <40 copies/ml at the time of assessment, while four were not on ART with median 45,708.8 copies/ml at the time of assessment. Median absolute CD4^+^ T-cell count from blood was 260 ​cells/microliter while median CD4^+^ T-cell percentage was 24%. Mean NPT-9 was 49.5 (standard deviation ​= ​8.1).

**Table 1:** Demographic/disease characteristics of participants.

PID	Age (years)	Sex	Race	ARV Therapy	Nadir CD4	CD4 Count	CSF WBC	CSF RBC	Plasma HIV log10	CSF HIV log10	NPT-9
124	54	F	AA	BIC, TAF, FTC	82	411	2	120	<1.60	<1.60	41.89
127	47	M	AA	FTC/TDF, DRV, COBI	NA	1434	5	1	<1.60	<1.60	42.00
130	58	MtF	AA	DTG, ABC, 3 ​TC	22	364	1	2	<1.60	<1.60	57.67
132	56	M	AA	ATV, COBI, TAF, FTC	18	194	0	0	<1.60	<1.60	55.89
138	47	M	W	BIC, TAF, 3 ​TC	11	730	0	0	<1.60	<1.60	50.22
139	68	M	W	DTG, ABC, 3 ​TC	NA	245	2	2	<1.60	<1.60	42.33
142	40	M	AA	TAF, BIC, FTC^a^	81	325	0	13	4.68	3.21	56.11
711	36	M	AA	Naïve	13	51	0	0	4.64	1.61	51.77
717	53	F	AA	Naive	89	89	0	0	5.68	4.22	36.99
733	55	M	W	Naïve	153	153	0	0	3.68	3.13	47.44
746	50	M	AA	FTC/TDF, FPV, RTV	9	260	0	0	<1.60	<1.60	62.70

Nadir CD4, CD4 Count, WBC, and RBC are reported in cells/microliter. PID ​= ​patient identification; ARV ​= ​anti-retroviral; BIC ​= ​bictegravir 50 ​mg once daily; TAF ​= ​tenofovir alafenamide 10–25 ​mg once daily; FTC ​= ​emtricitabine 200 ​mg once daily; FTC/TDF ​= ​Truvada (emtricitabine/tenofovir disoproxil fumarate) 1200–300 ​mg tablet once daily; DRV ​= ​darunavir 800 ​mg once daily; COBI ​= ​cobicistat 150 ​mg once daily; DTG ​= ​dolutegravir 50 ​mg once daily; ABC ​= ​abacavir 600 ​mg once daily; 3 ​TC ​= ​lamivudine 300 ​mg once daily; ATV ​= ​atazanavir 300 ​mg once daily; RTV ​= ​ritonavir 100 ​mg once-twice daily; FPV ​= ​fosamprenavir 700 ​mg twice daily; M ​= ​male; F ​= ​female; MtF ​= ​transgender male to female; AA ​= ​African American; W = White; CSF ​= ​cerebrospinal fluid; WBC ​= ​white blood cell; RBC ​= ​red blood cell; NPT-9 ​= ​neuropsychological composite global T score.

a Denotes PWH recently (<6 months) began ARV therapy and was not virally suppressed at time of draw.

**Fig. 2:**
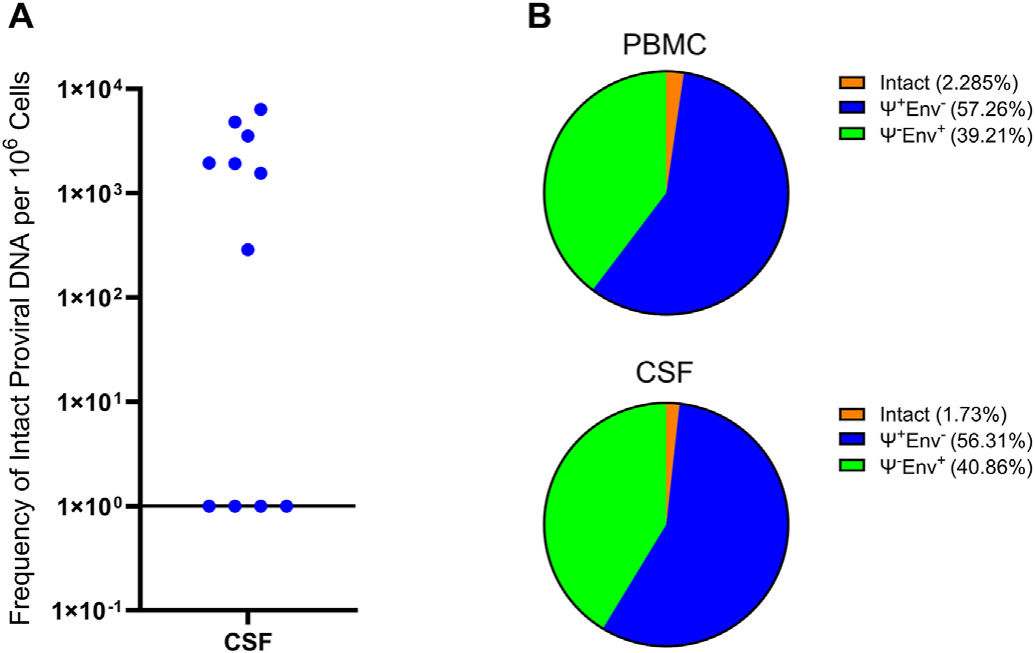
A) Intact proviral HIV DNA was detected in 7 of 11 people with HIV (PWH) in the cerebrospinal fluid (CSF) with a median of 1950 copies/10^6^ ​cells (IQR ​= ​287–6370). B) Median percentages of intact (peripheral blood mononuclear cells (PBMC) ​= ​2.285%, CSF ​= ​1.73%), Ψ+env- (PBMC ​= ​57.26%, CSF ​= ​56.31%), and Ψ-env ​+ ​HIV DNA (PBMC ​= ​39.21%, CSF ​= ​40.86%) were similar between PBMC from blood (top) and CSF (bottom).

**Fig. 3:**
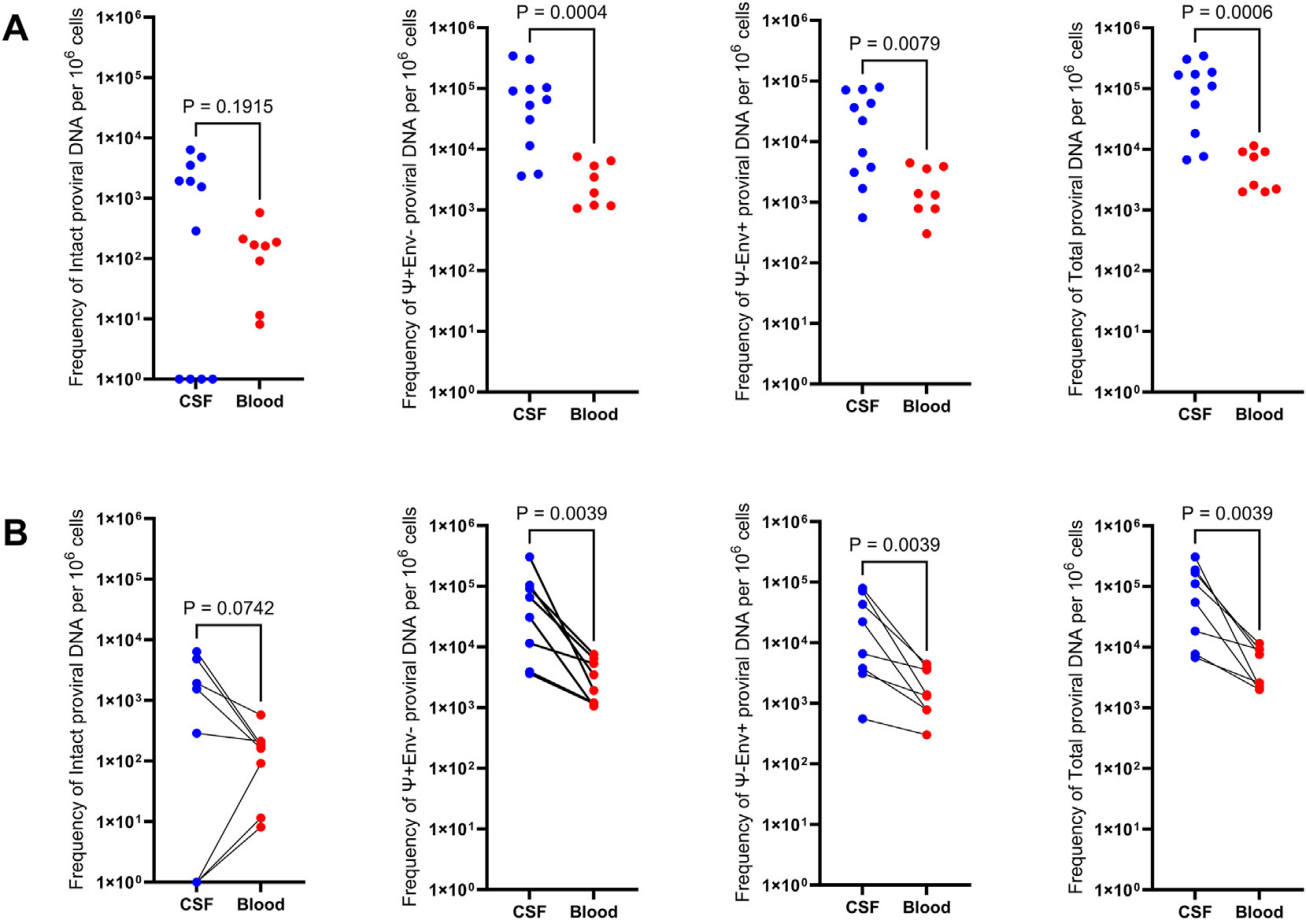
Frequencies of total, 3′ defective (Ψ+env-), and 5′ defective (Ψ-env+) proviral HIV DNA were all significantly (p ​< ​0.05) higher in cerebrospinal fluid (CSF) than peripheral blood mononuclear cells (PBMC) in both A) unmatched pairs (CSF n ​= ​11, PBMC n ​= ​8) and B) matched pairs (n ​= ​8). Intact proviral DNA was trending toward higher in CSF than PBMC in matched pairs (0.05<p ​< ​0.1). Wilcoxon signed rank for unmatched pairs and Wilcoxon signed rank for matched pairs, one-tailed, comparisons performed with GraphPad Prism v10.0.2, α ​= ​0.05.

Of the 11 participants, eight had matching PBMC for analysis. Total HIV DNA was detected in all CSF and PBMC specimens (Table 2). For the eight matched pairs, median total CSF HIV DNA was 8.29 ​× ​10^4^ copies per 10^6^ ​cells (Interquartile range [IQR] ​= ​6.75 ​× ​10^3^ – 3.06 ​× ​10^5^). This was significantly higher (P ​= ​0.0039) than in blood (median ​= ​5.06 ​× ​10^3^, IQR ​= ​2.00 ​× ​10^3^ – 1.15 ​× ​10^4^). This total HIV DNA difference remained significant compared to PBMC (P ​= ​0.0006) when all 11 CSF samples were included (median ​= ​1.11 ​× ​10^5^, IQR ​= ​6.75 ​× ​10^3^– 3.47 ​× ​10^5^). Intact HIV IPDA was detected in seven of 11 CSF samples (median ​= ​1950 copies/10^6^ ​cells, IQR ​= ​287–6370) (Fig. 2A) and in eight of eight PBMC samples (median ​= ​165.5 copies/10^6^ PBMC, IQR ​= ​8.31–578) (P ​= ​0.1 for comparison of detectability). Overall, the median proportions of intact (PBMC ​= ​2.285%, CSF ​= ​1.73%), Ψ+env- (PBMC ​= ​57.26%, CSF ​= ​56.31%), and Ψ-env ​+ ​HIV DNA (PBMC ​= ​39.21%, CSF ​= ​40.86%) were similar between compartments (Fig. 2B). For the eight matched pairs, median CSF intact IPDA was 9.19 ​× ​10^2^ per 10^6^ ​cells (IQR ​= ​0–6.37 ​× ​10^3^) while median blood intact IPDA was 1.66 ​× ​10^2^ per 10^6^ ​cells (IQR ​= ​8.13 ​× ​10° – 5.78 ​× ​10^2^), trending higher in CSF (P ​= ​0.0742). However, when all CSF samples were included, the median CSF IPDA was 1.55 ​× ​10^3^ (IQR ​= ​0–6.37 ​× ​10^3^), resulting in no significant difference from PBMC. In matched pairs, 3′ defective (Ψ+env-) (median ​= ​4.84 ​× ​10^4^; IQR ​= ​3.64 ​× ​10^3^ – 3.05 ​× ​10^5^) and 5′ defective (Ψ-env+) (median ​= ​4.45 ​× ​10^4^; IQR ​= ​558–7.93 ​× ​10^4^) HIV DNA in the CSF was significantly higher than 3′ defective (median ​= ​2.70 ​× ​10^3^; IQR ​= ​1.06 ​× ​10^3^ – 7.57 ​× ​10^3^) and 5′ defective (median ​= ​1.36 ​× ​10^3^; IQR ​= ​302–4.47 ​× ​10^3^) HIV DNA in PBMC (P ​= ​0.0039, 0.0039 respectively). This observation also occurred in unmatched pairs where 3′ defective (median ​= ​6.59 ​× ​10^4^; IQR ​= ​3.64 ​× ​10^3^ – 3.45 ​× ​10^5^) and 5′ defective (median ​= ​2.23 ​× ​10^4^; IQR ​= ​558–7.93 ​× ​10^4^) HIV DNA in the CSF was significantly higher than 3′ defective (median ​= ​2.70 ​× ​10^3^; IQR ​= ​1.06 ​× ​10^3^ – 7.57 ​× ​10^3^) and 5′ defective (median ​= ​1.36 ​× ​10^3^; IQR ​= ​302–4.47 ​× ​10^3^) HIV DNA in PBMC (P ​= ​0.0004, 0.0079 respectively). Frequency of 3′ defective, 5’ defective, and total HIV DNA were all significantly higher in CSF than PBMC in both unmatched (Wilcoxon rank sum, one-tailed, α ​= ​0.05) and matched pairs (Wilcoxon signed rank for matched pairs, one-tailed, α ​= ​0.05) (Fig. 3).

**Table 2:** Reservoir (HIV DNA) measurements in copies/10^6^ ​cells.

Participant ID	CSF Total HIV DNA	CSF Intact HIV DNA	CSF 3′Defective HIV DNA	CSF 5′Defective HIV DNA	CSF% Intact	Plasma Total HIV DNA	Plasma Intact HIV DNA	Plasma 3′Defective HIV DNA	Plasma 5′Defective HIV DNA	Plasma % Intact
124	1.88E+05	4.81E+03	1.04E+05	7.93E+04	2.55%	7.54E+03	1.69E+02	3.48E+03	3.90E+03	2.25%
127	6.75E+03	ND	3.64E+03	3.11E+03	0.00%	2.58E+03	9.16E+01	1.17E+03	1.32E+03	3.55%
130	5.47E+04	1.55E+03	3.08E+04	2.23E+04	2.83%	2.00E+03	1.62E+02	1.06E+03	7.85E+02	8.06%
132	1.69E+05	6.37E+03	9.12E+04	7.12E+04	3.77%	9.15E+03	1.88E+02	7.57E+03	1.39E+03	2.05%
138	1.84E+04	2.87E+02	1.15E+04	6.61E+03	1.56%	9.13E+03	2.12E+02	5.33E+03	3.59E+03	2.32%
139	1.11E+05	1.92E+03	6.59E+04	4.32E+04	1.73%	1.15E+04	5.78E+02	6.46E+03	4.47E+03	5.02%
142	7.67E+03	ND	3.89E+03	3.78E+03	0.00%	2.00E+03	8.13E+00	1.20E+03	7.90E+02	0.41%
711	3.06E+05	ND	3.05E+05	5.58E+02	0.00%	2.23E+03	1.15E+01	1.92E+03	3.02E+02	0.52%
717	3.47E+05	ND	3.45E+05	1.68E+03	0.00%					
733	1.74E+05	3.54E+03	9.74E+04	7.27E+04	2.04%					
746	9.19E+04	1.95E+03	5.32E+04	3.67E+04	2.12%					

ND ​= ​not detected (0 copies); CSF ​= ​cerebrospinal fluid.

**Fig. 4:**
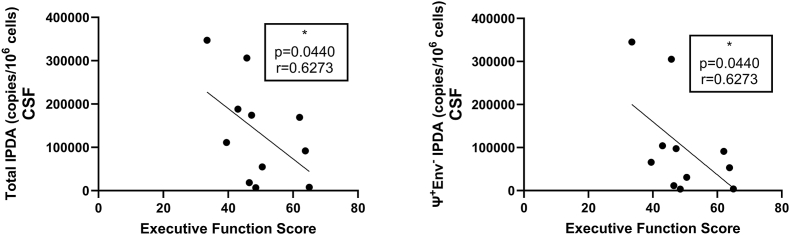
Executive function scores with practice in virally suppressed people with HIV negatively correlate to both total and 3′ defective HIV proviral DNA copies per 10^6^ ​cells in cerebrospinal fluid (CSF) (determined via intact proviral DNA assay (IPDA)). Correlations performed via Spearman's rho using Graphpad Prism v10.0.2 (α ​= ​0.05).

Some but not all HIV DNA results correlated between blood and CSF. Specifically, intact IPDA correlated significantly between the two compartments (rho ​= ​0.7075, P ​= ​0.032), as did 5′ defective HIV DNA (rho ​= ​0.6905, P ​= ​0.0347). Meanwhile, neither 3′ defective HIV DNA (rho ​= ​0.4286, P ​= ​0.1496) nor total HIV DNA (rho ​= ​0.2395, P ​= ​0.2820) correlated significantly between compartments. With respect to virologic suppression, total HIV DNA tended to be higher (P ​= ​0.0547 for CSF and P ​= ​0.0705 for blood) in those virologically suppressed on ART versus those not receiving ART. 3′ defective CSF HIV DNA levels were significantly lower in those on ART versus not receiving ART (P ​= ​0.0284). For neuropsychological performance, global and domains were examined. There was no significant difference in NPT-9 for those with detectable CSF IPDA versus undetectable CSF IPDA (P ​= ​0.5). There was no significant correlation between NPT-9 and levels of CSF HIV DNA (intact or defective, all P ​> ​0.2). However, higher CSF total HIV DNA tended to correlate with worse executive function. Specifically, a lower executive function T score was associated with both higher CSF 3’ defective HIV DNA (rho ​= ​−0.63, P ​= ​0.044) and higher CSF total HIV DNA (rho ​= ​−0.63, P ​= ​0.044), (Fig. 4).

## Discussion

Despite the successes of ART, HIV cure remains extremely difficult to achieve. HIV persists in the CNS, and clinical complications such as HIV-associated neurocognitive disorder (HAND) continue to occur [33]. Therefore, more research is needed to better understand the HIV CNS reservoir. The CSF is the most accessible part of the CNS. Previous studies have shown that HIV DNA is frequently detected from CSF cells and is associated with deleterious consequences such as cognitive impairment and neuronal damage [20,22]. However, previously published studies did not measure IPDA in CSF. Therefore, it was previously unclear if HIV DNA in CSF might be intact. The advent of IPDA has advanced HIV persistence research by offering scalability and accessibility advantages over existing approaches. Quantitative Viral Outgrowth Assay (QVOA) measures viral RNA and protein expression in latently infected T cells, but this assay is labor-intensive and requires significant cell numbers [34]. QVOA also may underestimate the size of the replication-competent virus reservoir, as single round T-cell stimulation does not reactivate the majority of latent proviruses *in vitro*.

The intact HIV proviral DNA assay (IPDA) was developed due to these logistical challenges of the QVOA [31]. Briefly, IPDA uses ddPCR and targets two regions of provirus: the Packaging Signal [Ψ] near the 5′ end of the viral genome and the Rev Responsive Element (RRE) within the envelope (*env*) gene toward the 3’ end. The assay defines intact proviruses by a lack of fatal defects, specifically large deletions in the 50 region of Ψ as well as APOBEC3G-mediated hypermutations or deletions in the RRE. This distinguishes genomically intact proviral sequences from defective ones.

Analysis of near full genome sequencing (nFGS) data in a previous study revealed that strategically positioned amplicons in the packaging signal (Ψ) and env regions collectively identified over 90% of deleted proviruses as defective. Identifying hypermutated proviruses is also crucial. Among these, 73% exhibit mutations in the GG→AG context. Additionally, most show GA→AA mutations. Only 27% of hypermutated proviruses exclusively display GA→AA mutations, with many also featuring deletions. Consequently, attention shifted to GG→AG hypermutation. Researchers pinpointed a conserved region in the RRE with neighboring consensus sites (TGGG) for the enzyme APOBEC3G. Among sequences with GG→AG hypermutation, 97% showcase one or more mutations in this region, spanning 13 distinct patterns. By utilizing mutant plasmids carrying each pattern, they developed allelic discrimination probes that accurately identify 95% of hypermutated sequences as defective [31].

A recent study by Brad Jones's group highlighted that HIV sequence polymorphism might contribute to IPDA detection failure. To overcome this challenge, the group devised a secondary set of primers/probes to accommodate the diversity in HIV sequences. However, incorporating more than two sets of primers/probes into the current QX200 Droplet Digital PCR system, which only features two fluorescence channels (FAM and HEX), poses a significant challenge due to the complexity of multiplexed digital PCR assays [32].

The polymorphism results in oligonucleotide-template mismatches, leading to reduced fluorescent amplitude on 2D scatter plots. Nevertheless, in certain instances, specific mismatches can be tolerated. Unlike real-time PCR, which tracks the effectiveness of each amplification cycle, digital droplet PCR primarily focuses on generating quantitative data at the end-point of PCR amplification. Consequently, even lower amplitude signals can still be considered positive, provided they adequately separate from the negative cluster.

We acknowledge the necessity to conduct a comprehensive analysis covering both 5′ and 3’ hypermutation and deletion. Notably, analysis of nFGS data indicated a notably low frequency (<3.8%) of proviruses carrying defective DNA sequences on both IPDA-targeted amplicons (Ψ-env-).

In multiple studies, IPDA results from blood correlated significantly with QVOA results from blood [31,35]. In the current study, our group used the IPDA to identify HIV DNA from cryopreserved CSF cell pellets and to differentiate intact versus defective virus. CSF HIV DNA was detectable in all samples. While this yield is higher than some published studies [20,21], newer technologies have yielded detectable CSF HIV DNA in upwards of 80% of PWH on ART [22], meaning that this may become a more common finding with the development and refinement of technology. We acknowledge that some of the participants in the current study were either not on ART or were on ART <2 years. Multiple studies have shown that the HIV reservoir in the blood continues to decay years after ART initiation, including studies identifying slow decay in intact HIV DNA [[35], [36], [37]]. The same may be true for the CNS, such that our results may have been different if all participants were on suppressive ART and for a longer period of time. We found that total HIV DNA levels were higher in CSF compared to blood. This finding is consistent with other studies [20,22] and may be due to the fact that CSF lymphocytes appear more likely to be positive for CCR5, the major co-receptor for HIV [22]. Therefore, these lymphocytes may be more susceptible to HIV infection and the establishment of viral latency. In addition, most antiretrovirals have >10-fold less penetration into the brain, resulting in a sanctuary for HIV [38]. Though not statistically significant, we also found that IPDA was slightly less likely to be detected from CSF than from blood, but these levels tended to be higher from CSF than blood when detectable. These findings may be multifactorial. One reason for the lower detectability is the difference in sheer abundance of lymphocytes and other HIV-susceptible mononuclear cells in CSF compared to blood in PWH [39]. Also, the vast majority of lymphocytes in general do not harbor intact HIV [40]. Thus, there are fewer scenarios in which latently infected lymphocytes would be found. Notably, limiting dilution assays have validated that quantitation of HIV DNA may not be substantially distorted by the low numbers of cells analyzed [10,20]. However, the amount of IPDA per cell might actually be higher in CSF given the enrichment of the CCR5 co-receptor among CSF lymphocytes. CSF lymphocytes in PWH are also more likely to be CXCR3+, especially in viremic individuals, again possibly increasing their susceptibility to HIV infection [41,42]. CSF lymphocytes (and potentially monocytes and microglia-like cells that have been found in CSF) have many other differences compared to blood lymphocytes based on single cell transcriptomics [43,44].

**Fig. 5:**
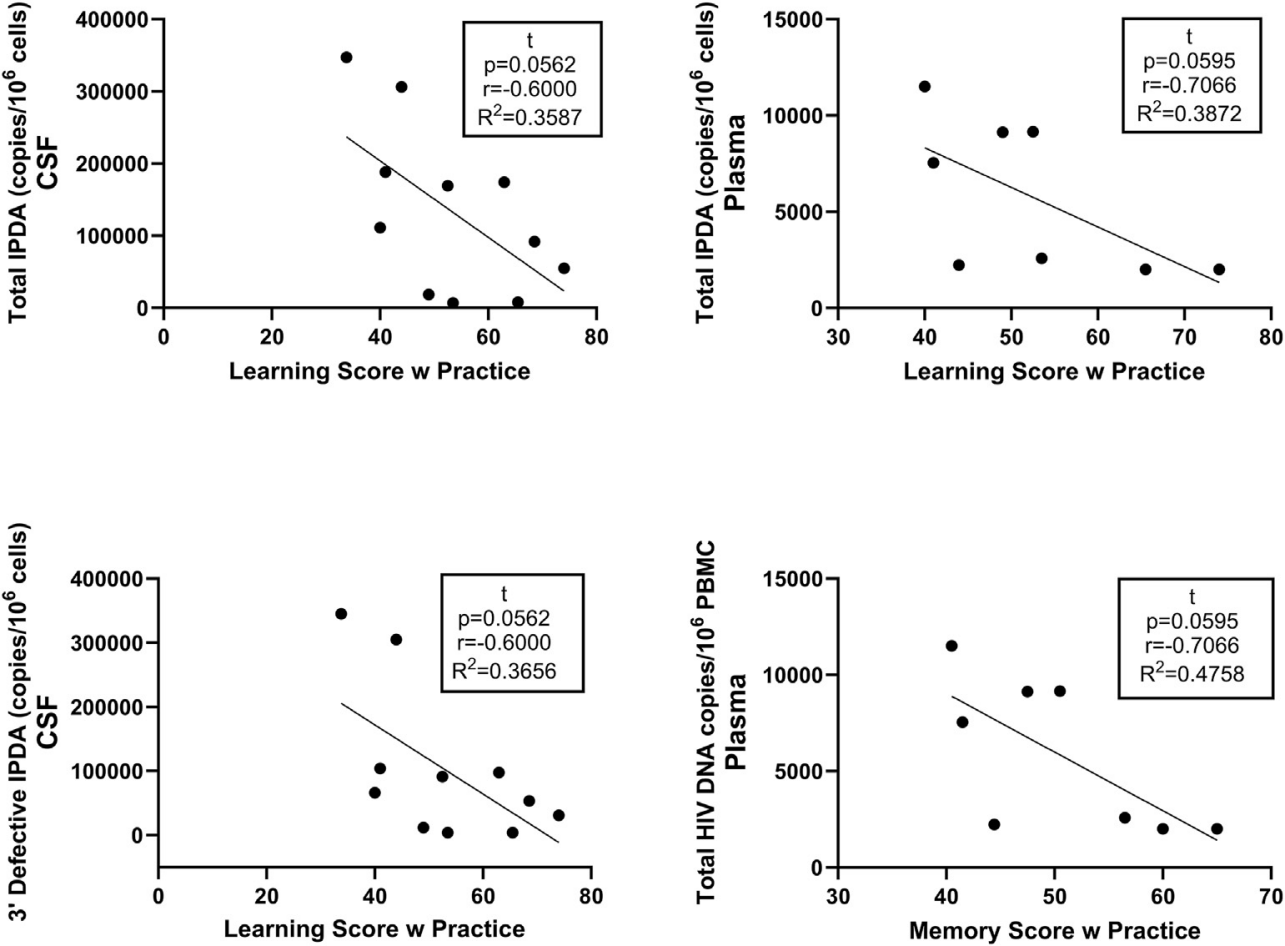
Learning scores with practice in virally suppressed people with HIV (PWH) trend (p ​= ​0.0562) toward negative correlation to both total and 3′ defective HIV proviral DNA copies per 10^6^ ​cells in cerebrospinal fluid (CSF) (determined via intact proviral DNA assay (IPDA)). Learning and memory scores with practice in virally suppressed PWH trend (p ​= ​0.0595) toward negative correlation to total HIV proviral DNA copies per 10^6^ peripheral blood mononuclear cells (PBMC). Correlations performed via Spearman's rho using GraphPad Prism v10.0.2 (α ​= ​0.05).

**Fig. 6:**
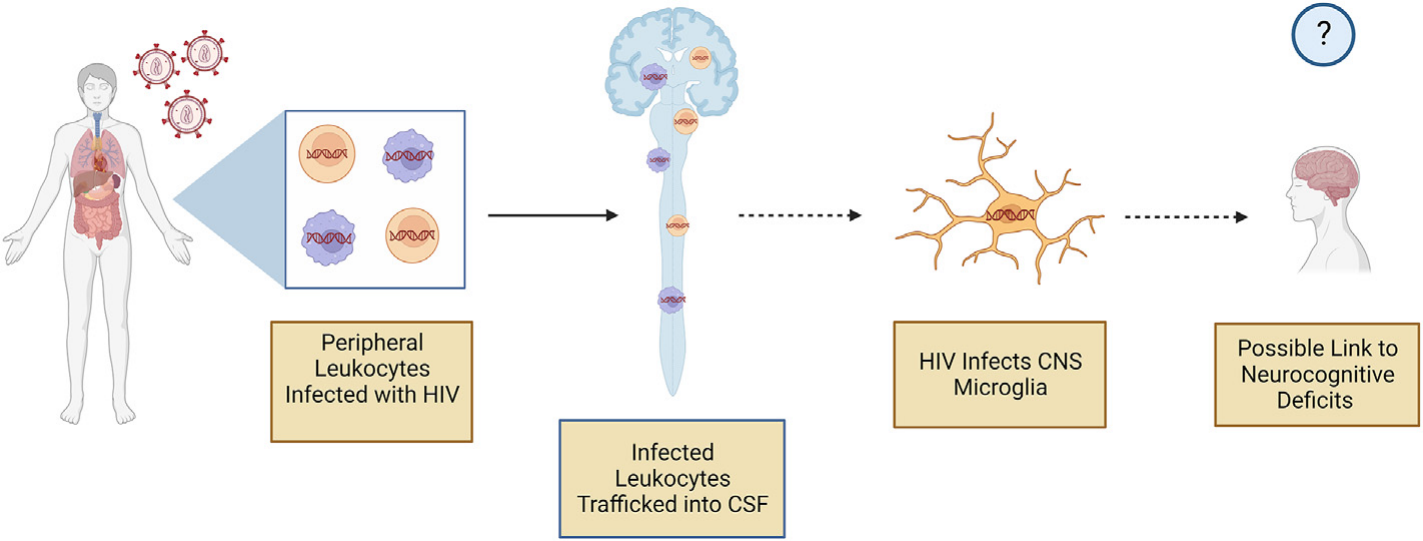
Proposed trafficking of peripherally infected cells into the central nervous system (CNS), infecting resident CNS cells and establishing the CNS as a viral reservoir which may lead to the development of neurocognitive deficits. Made with BioRender

We acknowledge that the current study had a small number of participants, and it is possible that the results may have differed with a larger cohort. We did not find significant associations with overall cognition (NPT-9), but the study was likely underpowered to detect significant associations in this regard. We did find a statistically significant correlation between higher 3′ defective HIV DNA in the CSF and worse executive function. While not statistically significant (P ​= ​0.0562), we also observed a negative correlation between higher total and 3′ defective HIV DNA in the CSF and learning scores. In comparison, we observed non-statistically significant (P ​= ​0.0595) negative correlations between learning and memory scores and total HIV DNA copies per million PBMC in the plasma (Fig. 5), highlighting a potential link between HIV in the periphery and CNS. It is possible that peripherally infected cells traffic into the CNS, infecting resident CNS cells to establish a viral reservoir that contributes to neurocognitive deficits (Fig. 6). Given that defective HIV DNA can still be the source of viral proteins [45], CNS HIV DNA may not need to be intact to be detrimental to cognition and other CNS functions. Lastly, the finding that intact and 5’ defective CSF HIV DNA levels were higher among participants with virologic suppression is surprising. However, HIV reservoir size varies widely among PWH [46]. Therefore, the current study may simply reflect that the participants on ART happened to have larger HIV reservoirs. Interestingly, the study of IPDA in human brain tissue showed that levels of intact HIV DNA tended to be higher in individuals on ART [10].

We acknowledge that IPDA has limitations as well. Recent studies have demonstrated that HIV sequence polymorphism may cause IPDA detection failure [32]. To address the detection failure, groups have designed secondary primers/probes to rescue the HIV sequence diversity. Due to the complexity of multiplexed digital PCR assays, however, introducing more than two sets of primers/probes to the current QX200 Droplet Digital PCR system can be challenging since the instrument only contains two fluorescence channels (FAM and HEX). The polymorphism causes oligonucleotides-template mismatch, which leads to reduced fluorescent amplitude on 2D scatter plots. However, in some cases, certain mismatches can be tolerated. Instead of tracking the effectiveness of each amplification cycle from real-time PCR, digital droplet PCR focuses on generating quantitative data at the endpoint of PCR amplification. Therefore, the lower amplitude signal can still be scored as positive, provided it sufficiently partitions from the negative cluster. Other refinements of the approach may be beneficial. One potential refinement will be the use of high-throughput CSF techniques such as fluorescence-activated cell sorting (FACS) [39,47]. This may allow for the identification of small cellular subsets that harbor the highest frequency of IPDA.

The current study provides further evidence that the CNS is an HIV reservoir and provides a rationale for larger studies comparing replication competent virus across compartments to fully elucidate the role of each compartment and its contribution to neurocognitive impairment in PWH.

## Funding

The authors gratefully acknowledge funding from the National Institute on Aging (R01AG062387 to AMA), the National Institute of Mental Health (R01MH128872 to AMA; R01MH128158 to AMA, CG, WT, and VCM), Anti microbial Resistance and Therapeutic Discovery Training Program, NIH grant number 5T32AI106699, as well as the Emory Center for AIDS Research (P30AI050409 to CG). Our funding sources had no involvement in study design, collection, analysis, or interpretation of data, writing of the report, or decision to submit the article for publication.

## Author contributions

**ZZ** conducted IPDA measurement and statistical analysis, **MDR** conducted correlative and statistical analysis, **ZZ** and **MDR** contributed equally to manuscript writing, editing, figure creation and literature investigation. **SR** contributed to manuscript writing, literature investigation, and figure creation. **VCM** and **WT** provided conceptual contributions. **DRF** and **SLL** conducted assessment of NP tests and computation of NP scores. **AMA** conducted sample (CSF and PBMC) collection and NP tests with participants, preliminary statistical analysis, manuscript writing, and provided conceptual contribution. **CG** performed conceptual contribution as well as manuscript writing and editing.

## Declaration of competing interest

VCM has received investigator-initiated research grants (to the institution) and consultation fees from Eli Lilly, Bayer, Gilead Sciences, and ViiV. The other authors had nothing to declare.
