# Supplementary material for: The identification of intact HIV proviral DNA from human cerebrospinal fluid
Source: Neurotherapeutics. 2024 May 14;21(4):e00373. doi: 10.1016/j.neurot.2024.e00373 (PMC11284557; doi:10.1016/j.neurot.2024.e00373)
Supplement: Multimedia component 1 [file mmc1.docx]

**Table S1** Primer and probe sequences for intact proviral DNA (IPDA) assay

| Prime/Probe Name | | Sequence | Fluorophore | Quencher |
| --- | --- | --- | --- | --- |
| IPDA Ψ | Ψ F | CAGGACTCGGCTTGCTGAAG | N/A | N/A |
|  | Ψ R | GCACCCATCTCTCTCCTTCTAGC | N/A | N/A |
|  | Ψ intact probe | TTTTGGCGTACTCACCAGT | FAM | MGBNFQ |
| IPDA env | env F | AGTGGTGCAGAGAGAAAAAAGAGC | N/A | N/A |
|  | env R | GTCTGGCCTGTACCGTCAGC | N/A | N/A |
|  | env intact probe | CCTTGGGTTCTTGGGA | VIC | MGBNFQ |
|  | env hyper probe | CCTTAGGTTCTTAGGAGC | N/A | MGBNFQ |

F = forward; R = reverse; N/A = not available

**Table S2** Thermal cycling program for droplet digital polymerase chain reaction (ddPCR) reactions

| Temperature | Time | Cycle |
| --- | --- | --- |
| 95˚C | 10 minutes | X 1 |
| 94˚C | 30 seconds | X 40 |
| 58˚C | 1 minute |  |
| 98˚C | 10 minutes | X 1 |
| 12˚C | **∞** | |

**Table S3** Primer and probe sequences for human ribonuclear P protein subunit p30 (RPP30) gene

| Prime/Probe Name | | Sequence | Fluorophore | Quencher |
| --- | --- | --- | --- | --- |
| IPDA RPP30-1 | RPP30-1 F | CCATTTGCTGCTCCTTGGG | N/A | N/A |
|  | RPP30-1 R | CATGCAAAGGAGGAAGCCG | N/A | N/A |
|  | RPP30-1 probe | AAGGAGCAAGGTTCTATTGTAG | FAM | MGBNFQ |
| IPDA RPP30-2 | RPP30-2 F | GATTTGGACCTGCGAGCG | N/A | N/A |
|  | RPP30-2 R | GCGGCTGTCTCCACAAGT | N/A | N/A |
|  | RPP30-2 probe | CTGACCTGAAGGCTCT | VIC | MGBNFQ |

Used for DNA shearing index [DSI] correction of intact proviral DNA assay (IDPA) results. N/A denotes not available
